# Poly-L-Lactic Acid Filler Increases Adipogenesis and Adiponectin in Aged Subcutaneous Tissue

**DOI:** 10.3390/polym17131826

**Published:** 2025-06-30

**Authors:** Seyeon Oh, Nala Shin, Sang Ju Lee, Kuk Hui Son, Kyunghee Byun

**Affiliations:** 1 Functional Cellular Networks Laboratory, Lee Gil Ya Cancer and Diabetes Institute, Gachon University, Incheon 21999, Republic of Korea; 2 LIBON Inc., Incheon 22006, Republic of Korea; 3 Soonsoo Dermatology & Antiaging Center, Jeonju 54969, Republic of Korea; 4 Yonsei Star Skin & Laser Clinic, Seoul 03789, Republic of Korea; 5 Department of Thoracic and Cardiovascular Surgery, Gachon University Gil Medical Center, Gachon University, Incheon 21565, Republic of Korea; 6 Department of Anatomy & Cell Biology, College of Medicine, Gachon University, Incheon 21936, Republic of Korea; 7 Department of Health Sciences and Technology, Gachon Advanced Institute for Health & Sciences and Technology (GAIHST), Gachon University, Incheon 21999, Republic of Korea

**Keywords:** poly-L-lactic acid, adipogenesis, skin rejuvenation

## Abstract

Poly-L-lactic acid (PLLA) filler, which increases volume and collagen synthesis, is used for skin rejuvenation. Subcutaneous adipose tissue (SAT) contains precursors that differentiate into mature adipocytes that secrete adiponectin, which modulates SAT function and increases adipogenesis. During aging, adiponectin and precursor cell functions decrease, reducing adipogenesis and facial volume. Adiponectin also increases collagen synthesis by stimulating fibroblasts. After hydrogen peroxide treatment to induce senescent adipocytes (3T3-L1) and aged skin, follow-up PLLA treatment increased adipogenesis by stimulating the nuclear factor erythroid-2-related factor 2 (NRF2)/peroxisome proliferator-activated receptor gamma (PPARγ)/CCAAT/enhancer binding protein alpha (C/EBPα) pathway. This resulted in increased adiponectin secretion, which promoted collagen synthesis and mitigated the loss of SAT volume. In the senescent adipocyte, PLLA increased NRF2/PPARγ/C/EBPα, adipogenesis factors (fatty acid binding protein 4, lipoprotein lipase, and cluster of differentiation 36), lipogenesis factors (ATP citrate lyase, acetyl-CoA carboxylase, and fatty acid synthase), adiponectin, and lipid droplet size. Treatment of senescent fibroblasts with conditioned medium from PLLA-treated adipocytes increased collagen1 and 3 and decreased matrix metalloproteinase1 and 3 expressions. Similarly, PLLA increased NRF2/PPARγ/C/EBPα, adipogenesis, and lipogenesis factors in aged mouse SAT. Also, PLLA increased adiponectin and adipocyte numbers without hypertrophy and increased collagen accumulation and dermal thickness. In summary, PLLA increased adipogenesis and adiponectin, which increased the volume of SAT and collagen synthesis, thereby rejuvenating aged skin.

## 1. Introduction

The ability of adipocytes in the subcutaneous adipose tissue (SAT) to accumulate lipids decreases with age [1], resulting in thinning and wrinkling of the skin. Thus, there is increasing interest in adipose tissue modulation for skin rejuvenation [2]. Mitigating the loss of adipose tissue and facial volume during aging can result in fuller faces that look younger than their years [3,4]. Moreover, adipocytes secrete growth factors and cytokines, such as insulin-like growth factor, fibroblast growth factor, vascular endothelial growth factor, and adiponectin, which affect various skin cells [5]. In fact, adipocytes modulate the proliferation of dermal fibroblasts [6], and intradermal adipocytes recruit fibroblasts during wound healing [6]. Thus, subcutaneous adipocytes likely affect the skin via paracrine or endocrine mechanisms [7].

Autologous fat transplantation can restore lost facial volume; however, the absorption and retention of the fat are unpredictable, and there may be complications at the do-nor site, such as neurovascular injury or the formation of abscesses, nodules, or cysts [8,9]. To avoid such problems, injectable fillers such as hyaluronic acid (HA) or collagen have been widely used to augment facial volume [10] and increase adipogenesis [11]. The injection of biomaterials such as acellular adipose matrix results in adipogenesis and angio-genesis, increasing soft tissue volume [10]. Thus, injectable biomaterials that increase adipogenesis could replace autologous fat transplantation for skin rejuvenation.

The conversion of adipocyte precursor cells into mature adipocytes by adipogenesis requires (1) the generation of a preadipocyte from a mesenchymal stem cell and (2) the differentiation of the preadipocyte to produce a mature adipocyte [12]. There are early and late differentiation phases [13]. In the early phase, transcriptional factors increase, such as peroxisome proliferator-activated receptor gamma (PPARγ) and the CCAAT/enhancer binding protein alpha (C/EBPα) [14].

PPARγ increases the expression of adipogenic genes such as those that make cluster of differentiation 36 (CD36), fatty acid binding protein 4 (FABP4), and lipoprotein lipase (LPL), which are involved in the uptake and intracellular trafficking of lipids and the breakdown of plasma triglycerides (TGs) [15,16,17]. Those increases in gene expression lead to increased lipid storage in adipocytes [15,16,17,18]. PPARγ also increases the expression of lipogenic genes such as fatty acid synthase (FAS), ATP citrate lyase (ACL), and acetyl-CoA carboxylase 1 (ACC1) [19,20,21]. Also, the expression of adipogenic lipid storage genes and lipogenic genes increases during the late phase of adipocyte differentiation [13]. PPARγ is also important for maintaining the phenotype of mature adipocytes [22,23]. It decreases lipotoxicity by promoting lipid storage in the mature adipocytes and the terminal differentiation of preadipocytes [19].

Aging affects several aspects of adipocytes. The reduction in adipogenic transcription factors such as PPARγ and C/EBPα during aging reduces adipocyte size and capacity for lipid storage [24], and the terminal differentiation of preadipocytes to mature adipocytes also decreases during aging [25,26]. PPARγ promotes the synthesis of adiponectin [27], which is synthesized and secreted from mature adipocytes rather than preadipocytes [28]. However, the expression of adiponectin decreases in adipose tissue during aging [29]. Adiponectin affects various cells in the skin as well as adipocytes. It promotes the proliferation of keratinocytes [30] and increases collagen synthesis in the fibroblast by increasing the expression of HA synthase [31]. In addition, adiponectin reduces the ultraviolet radiation-induced increase in matrix metalloproteinase1 (MMP1) and MMP3 and in-creases collagen3 synthesis in the fibroblast [7].

Nuclear factor erythroid 2-related factor 2 (NRF2) increases the expression of C/EBPα and PPARγ [32,33]. Previously, we showed that poly-D,L-lactic acid (PDLLA) filler in-creases collagen synthesis by increasing NRF2 expression in the macrophage [34]. This increase in NRF2 stimulates the secretion of transforming growth factor-beta (TGF-β) from adipose-derived stem cells, which increases collagen in the aged animal skin [34]. The injectable dermal poly lactic acid (PLA) fillers have four subtypes of chiral molecules: poly-D-lactic acid, poly-L-lactic acid (PLLA), mesoPLA, and PDLLA, which are widely used to increase extracellular matrix (ECM) synthesis and facial volume [35,36,37,38,39,40].

As PDLLA increases NRF2 in aged skin [34], we hypothesized that PLLA would increase NRF2 expression in the SAT, leading to upregulation of C/EBPα and PPARγ, which then would enhance adipogenesis and expression of lipogenic factors. Increased adipogenesis increases the release of adiponectin, leading to collagen accumulation, decreased MMP, and rejuvenation of the aged skin. Moreover, more mature adipocytes could thicken adipose tissue by maintaining lipid deposits in the adipocytes, attenuating the loss of SAT volume. We evaluated our hypotheses using a model of hydrogen peroxide (H_2_O_2_)-induced senescence in cells in vitro and in aged animal skin.

## 2. Materials and Methods

### 2.1. PLLA Preparation

PLLA were obtained from the SACCI Bio Co. (Seoul, Republic of Korea). Briefly, the preparation of PLLA was as follows [41,42]; L-lactide (108 g, 750 mmol; Corbion, Amsterdam, Netherlands), stannous octoate (0.09 mL; Sigma-Aldrich, St. Louis, MO, USA), and 1-dodecanol (0.15 g, 0.8 mmol; Sigma-Aldrich) were added to a 1 L double-jacketed reactor at room temperature with the latter two compounds serving as the catalyst and initiator, respectively. The mixture was heated to 130 °C gradually over 1 h and stirred at that temperature for an additional 4 h under nitrogen condition. The resulting PLLA was collected via vacuum filtration and then dissolved in DCM. Unreacted components were removed by precipitation using ethanol. The purified polymer was redispersed in dichloromethane (99.5%; Samchun Chemical Co., Ltd., Seoul, Republic of Korea) at room temperature. While stirring the solution at 2000 rpm using a shear mixer, we slowly added a 1% polyvinyl alcohol (87–90% hydrolyzed, molecular weight 30,000–70,000; Sigma-Aldrich) solution dropwise using a glass pipette. The mixture was then filtered under vacuum at room temperature for 3 h and centrifuged at 3000 rpm for 10 min to separate the PLLA. The supernatant was discarded, and the residual polyvinyl alcohol associated with the PLLA was re-dispersed in 50 mL of distilled water and centrifuged again at 3000 rpm for 10 min. Finally, the purified PLLA polymer (Mn 50–70 kDa) was obtained by drying in an oven at 45 °C for 24 h, resulting in microspheres with particle sizes ranging from 50 to 70 µm.

### 2.2. In Vitro Model and Experimental Design

#### 2.2.1. Cell Culture

3T3-L1 preadipocytes (American Type Culture Collection, Manassas, VA, USA) were cultured in high-glucose Dulbecco’s Modified Eagle Medium (DMEM; Gibco-Thermo Fisher Scientific, Waltham, MA, USA) supplemented with 10% bovine calf serum (BCS; Gibco) and 1% penicillin/streptomycin (P/S; Welgene, Gyeongsan, Republic of Korea).

CCD-986Sk dermal fibroblasts (American Type Culture Collection) were cultured in Iscove’s Modified Dulbecco’s Medium (IMDM; Welgene) containing 10% fetal bovine serum (FBS; Gibco-Thermo Fisher Scientific) and 1% P/S. All cells were maintained at 37 °C in a humidified incubator with 5% CO_2_.

#### 2.2.2. Adipocyte Differentiation

3T3-L1 preadipocytes (8 × 10^4^ cells/well) were seeded in six-well plates (SPL Life Sciences, Pocheon, Republic of Korea) and cultured to full confluence. To stabilize confluence, the cells were maintained for an additional 2 days in preadipocyte expansion medium (PEM; DMEM supplemented with 10% BCS and 1% P/S) from post-induction day (PID)0 to PID2. Adipogenic differentiation was initiated at PID2 by replacing PEM with differentiation medium (DM) consisting of DMEM supplemented with 10% FBS, 1% P/S, 1.0 μM dexamethasone (G-Biosciences, a Geno Technology, Inc. brand, St. Louis, MO, USA), 0.5 mM 3-isobutyl-1-methylxanthine (IBMX; Sigma-Aldrich), and 1.0 μg/mL insulin (Sigma-Aldrich). After 2 days (PID2–PID4), the medium was replaced with adipocyte maintenance medium (AMM; DMEM containing 10% FBS, 1% P/S, and 1.0 μg/mL insulin) for 3 days (PID4–PID7). From PID7 onward, AMM without insulin was used, and the medium was changed every 2 days (2 times in total) [43].

#### 2.2.3. Induction of Cellular Senescence and PLLA Treatment

Based on the method described by Zoico, E. et al. [44], we modified the protocol to fit our experimental design. Cellular senescence was induced in 3T3-L1 preadipocytes by repeated treatment with 150 μM H_2_O_2_ (Sigma-Aldrich). Cells at PID2 were exposed to H_2_O_2_ for 3 h daily for three consecutive days (PID2–PID4). Following each treatment, cells were washed twice with Dulbecco’s phosphate-buffered saline (DPBS, Gibco-Thermo Fisher Scientific) and incubated in fresh medium–DM on PID2 and PID3 and AMM on PID4 [43]. On PID5, PLLA was applied to cultures (Figure S1).

The cytotoxicity of PLLA was evaluated at concentrations of 0–50 mg/mL, and the optimal treatment concentration was determined using 0–2 mg/mL PLLA. Subsequently, a final concentration of 1 mg/mL PLLA was used. Cells were maintained under standard adipogenic differentiation conditions until PID10, when samples were collected for analysis [43].

For further analysis of adipocyte–fibroblast interactions, conditioned medium (CM) was collected from senescent adipocytes. CM was clarified by centrifugation at 300× *g* for 5 min to remove cellular debris and used immediately or stored at −80 °C for later use. CCD-986Sk fibroblast cells were treated with 350 µM H_2_O_2_ for 1.5 h to induce cellular senescence. After treatment, cells were washed with DPBS and cultured in a fresh growth medium for 72 h [45]. Subsequently, fibroblasts were incubated with adipocyte-derived CM for 3 days and harvested at the end of treatment for further analysis (Figure S2).

### 2.3. In Vivo Model and Experimental Design

#### 2.3.1. Mouse Model and Maintenance

C57BL/6 mice (6 weeks old) were obtained from the Orient Bio (Seongnam, Republic of Korea) and acclimatized for one week under controlled environmental conditions (temperature, 20–24 °C, humidity, 45–55%) with ad libitum access to food and water. For the experiment, offspring born from in-house mating of the initially purchased mice were used. After acclimatization, these in-house-bred mice and their offspring were aged to 12–13 months to ensure sample collection at 14 months of age. All animal procedures were approved by the Gachon University Animal Experiment Ethics Committee (IACUC approval number: LCDI-2023-0106) and complied with the ethical principles of the Association for Assessment and Accreditation of Laboratory Animal Care International (AAALAC International, Frederick, MD, USA).

#### 2.3.2. Experimental Design and PLLA Injections

Female aged mice were randomly assigned to five experimental groups (*n* = 5 per group) [46]. Animals exhibiting abnormal body weight (<30 g or >40 g) or visible signs of tumor formation were excluded from the study. A total of 25 animals were used in the final analysis. The control group (Group 1) received a subcutaneous injection of 500 μL sterile saline into five separate sites within the back area (2 cm × 2 cm) using a 27-gauge needle, and groups 2–5 received PLLA (10 mg/mL). To synchronize tissue collection time points at 14 months of age, we staggered injections as follows:

Group 1: saline was injected at 12 months of age and sampled after 8 weeks

Group 2: PLLA was injected at 12 months of age and sampled after 8 weeks

Group 3: PLLA was injected at 13 months of age and sampled after 4 weeks

Group 4: PLLA was injected at 13.5 months of age and sampled after 2 weeks

Group 5: PLLA was injected at 13.8 months of age and sampled after 1 week.

Prior to tissue collection, skin elasticity was measured 10 times per mouse using a portable skin analyzer (API-100; Aram Huvis, Seongnam, Republic of Korea). Skin and SAT samples were collected at the indicated post-injection time points for analyses (Figure S3).

### 2.4. Cell Cytotoxicity

To evaluate PLLA cytotoxicity, senescent adipocytes (described in Section 2.2.3) were plated in a 24-well plate (SPL Life Sciences) and treated with increasing concentrations of PLLA (0–50 mg/mL) for 5 days. Cell viability was assessed using a Cell Counting Kit-8 (TransGen Biotech Co., Ltd., Beijing, China), and absorbance was measured at 450 nm. Experiments were performed in triplicate.

### 2.5. RNA Extraction, cDNA Synthesis, and Gene Expression Analysis

Total RNA was isolated from both adipocytes and SAT using RNAiso reagent (TAKARA, Tokyo, Japan) according to the manufacturer’s instructions. RNA concentration and purity were measured using a NanoDrop spectrophotometer (Thermo Fisher Scientific). For cDNA synthesis, 1 μg of total RNA was reverse-transcribed into complementary DNA using a commercial cDNA synthesis kit (TAKARA), following the provided protocol.

Quantitative reverse transcription–polymerase chain reaction (qRT-PCR) was performed on cDNA synthesized as described above using a SYBR Green premix (TAKARA) on a QuantStudio™ 3 Real-Time PCR System (Thermo Fisher Scientific). Thermal cycling conditions were as follows: initial denaturation at 95 °C for 10 min, followed by 40 cycles at 95 °C for 15 s, 60 °C for 1 min, and another 15 s at 95 °C. Gene expression levels were calculated using the ΔΔCt method and normalized to ACTB (β-actin) [47]. Primer sequences are listed in Table S1.

### 2.6. Protein Isolation and Analysis

#### 2.6.1. Protein Isolation and Quantitation

Proteins were isolated from cells and SAT using EzRIPA buffer (ATTO Corporation, Tokyo, Japan). Protein concentrations were determined using the bicinchoninic acid assay (Thermo Fisher Scientific). Lysates were either used immediately for downstream applications or stored at −80 °C.

#### 2.6.2. Measurement of Adiponectin

The level of adiponectin in CM of senescent adipocytes and mouse SAT was assessed using an Adiponectin ELISA kit (Mybiosource, Inc., San Diego, CA, USA) according to the manufacturer’s instructions.

#### 2.6.3. Western Blotting

Protein samples (30 μg) from cells or SAT samples were mixed with 4× lithium dodecyl sulfate sample buffer and 10× reducing reagent (Thermo Fisher Scientific), then denatured by heating at 70 °C for 10 min. Samples were loaded onto 10% sodium dodecyl sulfate–polyacrylamide gels and separated using MOPS running buffer (Invitrogen, Thermo Fisher Scientific, Waltham, MA, USA) at 200 V for 25 min. Proteins were transferred onto polyvinylidene fluoride membranes (Millipore, Burlington, MA, USA) using a semi-dry transfer system (ATTO) at 1 A for 10 min. Membranes were blocked in 5% skim milk (LPS solution, Daejeon, Republic of Korea) diluted in Tris-buffered saline with 0.1% Tween 20 (TTBS; SPL Life Sciences) for 1 h at room temperature on a shaker. After blocking, the membranes were washed three times with 0.1% TTBS and incubated overnight at 4 °C with primary antibodies diluted in 0.1% TTBS (Table S2). Following additional washes, membranes were incubated with horseradish peroxidase-conjugated secondary antibodies (1:5000; Vector Laboratories, Burlingame, CA, USA) for 1 h at room temperature. Protein bands were visualized using enhanced chemiluminescence substrates and imaged with a ChemiDoc imaging system (Bio-Rad, Hercules, CA, USA). Band intensities were quantified using ImageJ software (version 1.53s, NIH, Bethesda, MD, USA), using β-actin as a loading control. Expression levels were expressed relative to the control group, corresponding to the first bar in the graph shown for each comparison.

### 2.7. Staining

#### 2.7.1. Oil Red O Staining

At PID10, senescent adipocytes cultured in six-well plates (described in Section 2.2.3) were fixed in 10% formalin at room temperature for 30 min and then rinsed with distilled water. Fixed cells were incubated with propylene glycol for 5 min and stained with Oil Red O solution (Abcam, Cambridge, MA, USA) for 1 h at room temperature. Excess stain was removed with 85% propylene glycol, and cells were rinsed twice with distilled water. Nuclei were counterstained with hematoxylin for 10 s, rinsed with tap water, and mounted for microscopic imaging.

#### 2.7.2. Immunocytochemistry

Senescent fibroblasts cultured in eight-well chamber slides and treated with adipocyte-CM for 72 h (described in Section 2.2.3) were fixed in 10% formalin at room temperature for 30 min. Following three washes with phosphate-buffered saline (PBS), nonspecific binding was blocked using a serum-blocking solution (Vector Laboratories) for 1 h at room temperature. Cells were incubated overnight at 4 °C with primary antibodies against collagen1 and 3 (Table S2). Cells were washed with PBS and incubated with Alexa Fluor 488-conjugated secondary antibodies (1:500; Invitrogen) for 1 h at room temperature. Nuclei were counterstained with DAPI (1 μg/mL; Sigma-Aldrich) for 30 s, and slides were mounted using Vectashield antifade mounting medium (Vector Laboratories). Analysis was conducted using a confocal microscope (LSM-710; Carl Zeiss, Oberkochen, Germany) at the Core-facility for Cell to In-vivo imaging. Confocal images were captured from five randomly selected fields of view per group, and the mean fluorescence intensity was analyzed using ZEN software (Carl Zeiss).

#### 2.7.3. Fixation and Paraffin-Embedding of Skin Tissue

Skin tissue samples were fixed with 4% paraformaldehyde (Sigma-Aldrich) for 72 h, dehydrated, and embedded in paraffin using an automated tissue processor (Leica, Wetzlar, Germany). Paraffin blocks were sectioned into 7 µm slices using a microtome, mounted on glass slides, and incubated overnight at 60 °C to allow proper adhesion.

#### 2.7.4. Hematoxylin and Eosin Staining

Paraffin-embedded skin tissue sections were deparaffinized and rehydrated through sequential immersion in xylene and a graded ethanol series (100–70%). Deparaffinized slides were stained with hematoxylin (KPNT, Cheongju, Republic of Korea) for 1 min, rinsed, and then counterstained with eosin (KPNT) for 30 s. The stained slides were dehydrated, mounted, scanned using a slide scanner (Motic Scan Infinity 100; Motic, Beijing, China), and captured for analysis. Adipocyte size and number and SAT thickness were quantified using ImageJ software (version 1.53s, NIH).

#### 2.7.5. Immunohistochemistry (DAB, 3,3′-Diaminobenzidine)

Permeabilization was performed by incubating the sections in PBS containing 0.5% Triton X-100 for 5 min on deparaffinized slides. Following another three washes with PBS, nonspecific binding was blocked with a serum-blocking solution (Vector Laboratories) for 1 h at room temperature. The slides were then incubated overnight at 4 °C with primary antibodies (Table S2). After incubation, slides were washed with PBS and incubated with biotin-conjugated secondary antibodies (1:200; Vector Laboratories) for 1 h at room temperature. The slides were subsequently washed with PBS, incubated with the ABC reagent (Vector Laboratories), and washed again. Color development was achieved by incubating the sections in DAB solution (Sigma-Aldrich) for 30 s to produce a brown color reaction. For counterstaining, slides were incubated in hematoxylin (KPNT) for 30 s, washed with distilled water, dehydrated using graded ethanol, and mounted with DPX Mount Solution (Sigma-Aldrich). The stained tissues were scanned using a slide scanner (Motic Scan Infinity 100; Motic), and images were captured for analysis. To quantify protein expression, DAB-positive signals in the dermis were quantified using ImageJ software (version 1.53s, NIH). The area with positive DAB staining was manually selected based on color thresholding, and the mean intensity within the selected region was measured. Quantification values were obtained from five randomly selected dermal fields per group.

#### 2.7.6. Masson Trichrome Staining

Staining was performed using a commercial Trichrome Stain Kit Modified Masson (Scytek Laboratories, Logan, UT, USA) according to the manufacturer’s instructions. The protocol included pre-treatment with Bouin’s Fluid and sequential staining with Weigert’s iron hematoxylin, Biebrich scarlet-acid fuchsin solution, phosphomolybdic/phosphotungstic acid solution, aniline blue solution, and 1% acetic acid. Stained slides were dehydrated and mounted, scanned using a slide scanner (Motic Scan Infinity 100; Motic), and images were captured for analysis. Collagen fiber density was quantified using ImageJ software (version 1.53s, NIH).

#### 2.7.7. Herovici Staining

Staining was performed using a commercial Herovici Stain Kit (Scytek Laboratories, Logan, UT, USA) according to the manufacturer’s instructions. Slides were stained with Weigert’s iron hematoxylin followed by Herovici solution and mounted after dehydration. Stained slides were scanned using a slide scanner (Motic Scan Infinity 100; Motic), and images were captured for analysis. Newly synthesized collagen fiber and mature collagen fiber density were quantified using ImageJ software (version 1.53s, NIH).

### 2.8. Statistical Analysis

All experiments were repeated independently at least three times. Quantitative data are presented as mean ± standard deviation (SD). Statistical comparisons were performed using the Kruskal–Wallis test followed by the Mann–Whitney U test for post hoc analysis. A *p*-value < 0.05 was considered statistically significant. Analyses were performed using SPSS version 26 (IBM, Armonk, NY, USA).

## 3. Results

### 3.1. PLLA Increased Expression of NRF2 and Factors of Adipogenesis and Lipogenesis in Senescent Preadipocytes

The optimal concentration of PLLA for in vitro experiments was determined based on its effect on adipocyte viability and *NRF2* expression. Adipocyte precursors (3T3-L1) were differentiated into mature adipocytes [43]. To generate a senescent preadipocyte model that mimics the aging-associated decrease in adipogenesis, we administered H_2_O_2_ during the initial differentiation of preadipocyte (Figure S1).

**Figure 1:**
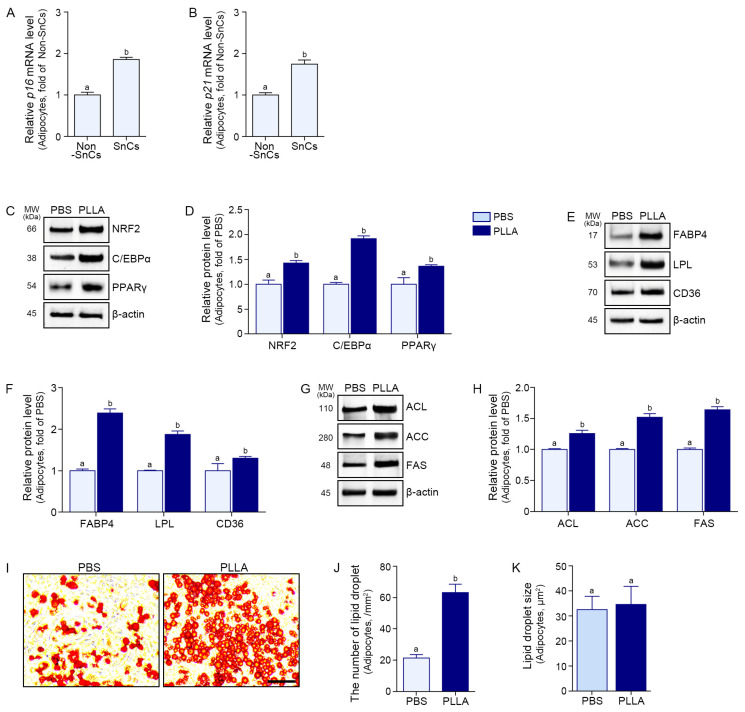
PLLA enhances adipogenesis and lipogenesis in senescent adipocytes. (**A**,**B**) qRT-PCR analysis of *p16* (**A**) and *p21* (**B**) mRNA expression levels in senescent adipocytes showed induction of cellular senescence. (**C**) Western blot analysis of adipogenic transcription factors, NRF2, C/EBPα, and PPARγ, in PBS- and PLLA-treated senescent adipocytes. β-actin was the loading control. (**D**) Quantification of protein expression in (**C**), normalized to β-actin and expressed as fold change relative to the PBS-treated senescent adipocyte. (**E**) Expression of adipogenic factors FABP4, LPL, and CD36 assessed by Western blots. (**F**) Protein levels in (**E**) were normalized to β-actin and shown as fold change relative to the PBS-treated senescent adipocyte. (**G**) Expression of lipogenesis-related factors ACL, ACC, and FAS assessed by Western blots. (**H**) Densitometric analysis of (**G**) was performed after normalization to β-actin, and results are presented as fold change compared to the PBS-treated senescent adipocyte. (**I**) Representative Oil Red O-stained images showing intracellular lipid droplet accumulation and droplet size in senescent adipocytes treated with PBS or PLLA (scale bar = 30 μm). (**J**,**K**) Quantitative analysis of the number of lipid droplets (**J**) and lipid droplet size (**K**) in senescent adipocytes as a measure from images shown in (**I**). All data are presented as mean ± SD from at least three independent experiments. Statistical significance was determined using the Kruskal–Wallis test followed by the Mann–Whitney U test. Bars labeled with different letters indicate statistically significant differences (*p* < 0.05). ACC, acetyl-CoA carboxylase; ACL, ATP citrate lyase; CD36, cluster of differentiation 36; C/EBPα, CCAAT/enhancer binding protein alpha; FABP4, fatty acid binding protein 4; FAS, fatty acid synthase; LPL, lipoprotein lipase; MW, molecular weight; Non-SnCs, non-senescent cells; NRF2, nuclear factor erythroid-2-related factor 2; PBS, phosphate-buffered saline; PLLA, poly-l-lactic acid; PPARγ, peroxisome proliferator-activated receptor gamma; SD, standard deviation; SnCs, senescent cells; qRT-PCR, quantitative reverse transcription polymerase chain reaction.

This treatment resulted in increased expression of *p16* and *p21*, widely used markers of senescence [48] (Figure 1A,B). Cell viability was determined after treatment with PLLA at 0 to 50 mg/mL. The viability of senescent adipocytes did not decrease significantly at PLLA concentrations up to 50 mg/mL (Figure S4A). Next, the optimal concentration of PLLA was determined based on an increased mRNA expression of *NRF2* in the senescent adipocyte. The expression of *NRF2* increased similarly with PLLA at 1 or 2 mg/mL (Figure S4B). Thus, 1 mg/mL PLLA was used in subsequent experiments.

In the senescent adipocyte, PLLA induced a significant increase of NRF2, C/EBPα, and PPARγ (Figure 1C,D). PLLA also increased adipogenic factors FABP4, LPL, and CD36 in the senescent adipocyte (Figure 1E,F). PLLA increased expression of lipogenesis-related factors ACL, ACC, and FAS (Figure 1G,H).

### 3.2. PLLA Increased Lipid Droplet and Adiponectin Secretion in Senescent Adipocytes

**Figure 2:**
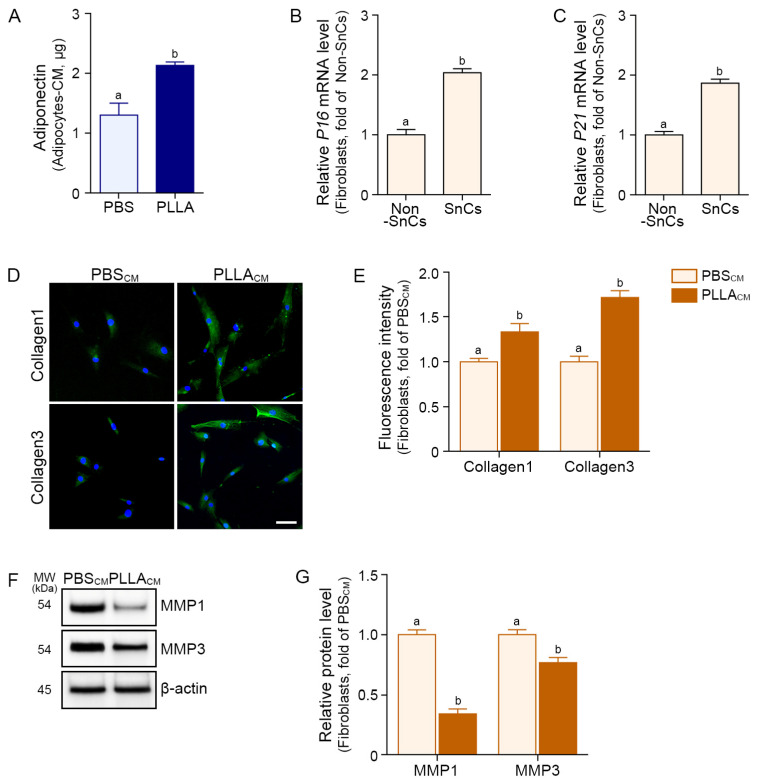
CM from PLLA-treated senescent adipocytes modulates collagen and MMP expression in senescent fibroblasts. (**A**) The adiponectin concentration in adipocyte-CM was measured by an ELISA following PBS or PLLA treatment. (**B**,**C**) qRT-PCR analysis of *P16* (**B**) and *P21* (**C**) mRNA expression levels in senescent fibroblasts showed induction of cellular senescence. (**D**) Representative immunofluorescence images showing collagen1 and collagen3 (green) expression in senescent fibroblasts cultured with PBS_CM_ or PLLA_CM_. Nuclei were counterstained with DAPI (blue). Scale bar = 50 μm. (**E**) Quantification of collagen1 and collagen3 mean fluorescence intensities from (**D**). Data were obtained from five randomly selected fields per group. (**F**) Western blot analysis of MMP1 and MMP3 in fibroblasts exposed to PBS_CM_ or PLLA_CM_. (**G**) Protein levels in (**F**) were normalized to β-actin and shown as fold change relative to the PBS_CM_-treated senescent fibroblast. All data represent mean ± SD from at least three independent experiments. Statistical significance was assessed using the Kruskal–Wallis test followed by the Mann–Whitney U test. Bars marked with different letters indicate statistically significant differences between groups (*p* < 0.05). CM, conditioned medium; DAPI, 4′,6-diamidino-2-phenylindole; ELISA, enzyme-linked immunosorbent assay; MMP, matrix metalloproteinases; MW, molecular weight; Non-SnCs, non-senescent cells; PBS, phosphate-buffered saline; PBS_CM_, CM from PBS-treated senescent adipocytes; PLLA, poly-l-lactic acid; PLLA_CM_, CM from PLLA-treated senescent adipocytes; SD, standard deviation; SnCs, senescent cells; qRT-PCR, quantitative reverse transcription polymerase chain reaction.

Mature adipocytes deposited lipids in the cytoplasm, as seen by Oil Red O staining (Figure 1I). PLLA increased the number of lipid droplets in adipocytes, but the droplet size remained unchanged (Figure 1J,K). Adiponectin secretion was also increased by PLLA treatment in the CM from senescent adipocyte (Figure 2A).

Fibroblast is the major cell that synthesizes collagen; hence, we hypothesized that adiponectin secreted by the mature adipocyte would promote increased collagen synthesis by fibroblasts. To evaluate this hypothesis, we administered CM from PBS- or PLLA-treated senescent adipocytes to senescent fibroblasts that were induced by H_2_O_2_ treatment (Figure S2) and confirmed by increased expression of the senescence markers p16 and p21 (Figure 2B,C). By treating CM from PLLA-treated senescent adipocytes (PLLA_CM_), expression of collagen1 and 3 were increased in the senescent fibroblast (Figure 2D,E). PLLA_CM_ decreased expression of MMP1 and 3 in the senescent fibroblast (Figure 2F,G).

### 3.3. PLLA Increased Expression of NRF2, Adipogenesis and Lipogenesis Factors, and Thickness of SAT in the Aged Animal Skin

**Figure 3:**
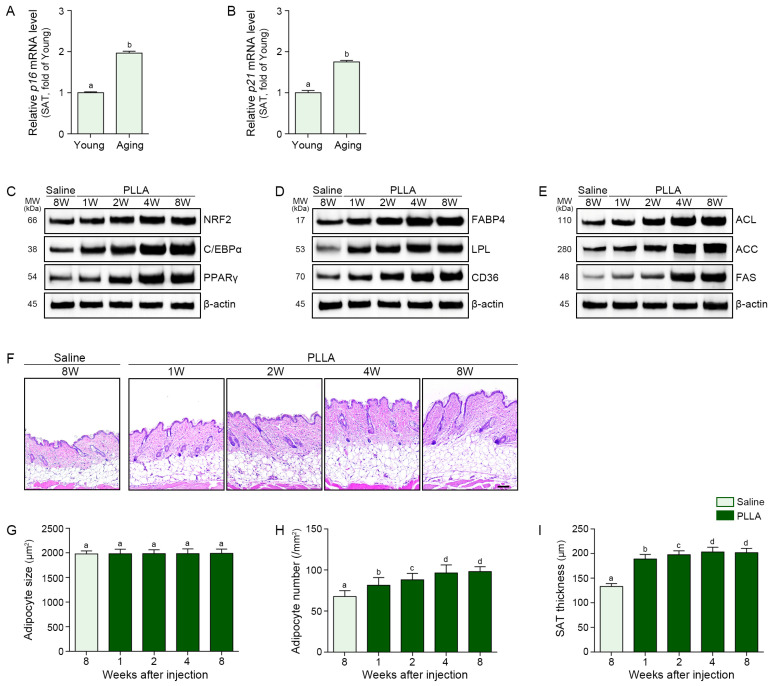
Effects of PLLA on adipogenesis and lipogenesis in the SAT of aged mice over time. (**A**,**B**) qRT-PCR analysis of *P16* (**A**) and *P21* (**B**) mRNA expression levels in SAT samples from aged mice, demonstrating the induction of cellular senescence. (**C**) Western blot analysis of adipogenic transcription factors NRF2, C/EBPα, and PPARγ. (**D**) Expression of adipogenic factors FABP4, LPL, and CD36. (**E**) Expression of lipogenesis-related factors ACL, ACC, and FAS. β-actin was used as a loading control for all blots. (**F**) Representative hematoxylin and eosin-stained images showing morphological changes and progressive thickening of the adipose layer following PLLA injection (scale bar = 100 μm). (**G**) Quantification of average adipocyte size. (**H**) Quantification of adipocyte number. (**I**) Measurement of total SAT thickness. All quantitative data represent mean ± SD from *n* = 5 animals per group. Statistical analysis was performed using the Kruskal–Wallis test followed by the Mann–Whitney U test for post hoc comparison. Bars labeled with different letters indicate statistically significant differences between groups (*p* < 0.05). ACC, acetyl-CoA carboxylase; ACL, ATP citrate lyase; CD36, cluster of differentiation 36; C/EBPα, CCAAT/enhancer binding protein alpha; FABP4, fatty acid binding protein 4; FAS, fatty acid synthase; LPL, lipoprotein lipase; MW, molecular weight; NRF2, nuclear factor erythroid-2-related factor 2; PLLA, poly-l-lactic acid; PPARγ, peroxisome proliferator-activated receptor gamma; SAT, subcutaneous adipose tissue; SD, standard deviation; qRT-PCR, quantitative reverse transcription polymerase chain reaction; W, weeks.

To assess the in vivo effects of PLLA, 12–14-month-old male mice were injected subcutaneously with PLLA (10 mg/mL) or saline, and skin and SAT samples were collected 1, 2, 4, and 8 weeks after injection for analysis (Figure S3). Expression of *p21* and *p16* increased in the SAT of aged skin (Figure 3A,B). PLLA induced significant increases in NRF2, C/EBPα, and PPARγ in the aged SAT (Figure 3C and Figure S5A–C). PLLA also increased the expression of FABP4, LPL, and CD36 in the aged SAT (Figure 3D and Figure S5D–F) as well as the expression of lipogenesis factor ACL, ACC, and FAS in the aged SAT (Figure 3E and Figure S5G–I). Although expression of all proteins increased with time after PLLA injection, expression levels between 4 and 8 weeks after PLLA injection were not significantly different (Figure 3C–E and Figure S5). Adipocyte size did not increase after PLLA injection in the aged SAT (Figure 3F,G); however, the number of adipocytes and the SAT thickness increased up to 4 weeks after PLLA injection (Figure 3F,H,I). In contrast, no significant changes were observed over time in the saline-injected group (Figure S6).

### 3.4. PLLA Increased Adiponectin and Collagen1/3 and Decreased MMP1/3 in the Aged Skin

**Figure 4:**
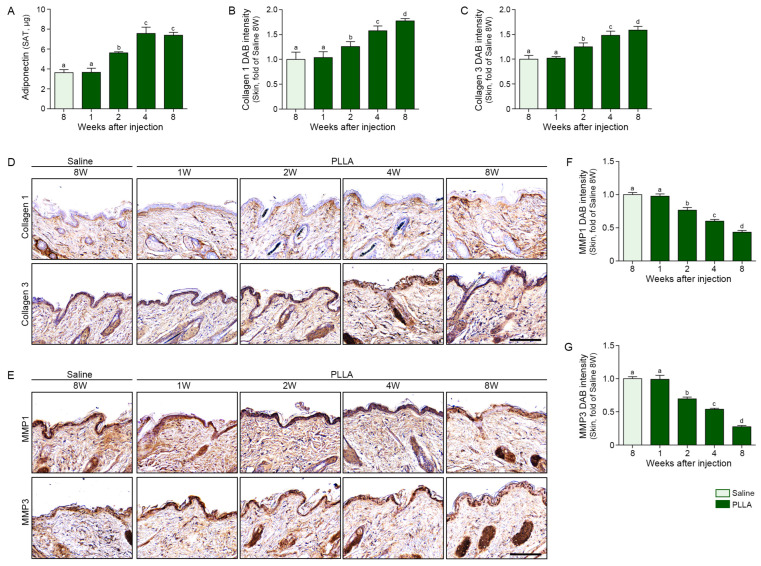
PLLA injection increases adiponectin and collagen expressions and reduces MMP levels in aged mouse skin. (**A**) Adiponectin concentrations in skin tissue were measured by ELISA. (**B**,**C**) Quantitative analysis of collagen1 (**B**) and 3 (**C**) expression based on mean DAB staining intensity from IHC image shown in (**D**). (**D**) Representative IHC images of collagen1 and 3 expressions in skin tissue (scale bar = 100 μm). (**E**) Representative IHC images of MMP1 and MMP3 expression (scale bar = 100 μm). Positive signals are shown in brown (DAB), and nuclei are counterstained in blue (hematoxylin). (**F**,**G**) Quantification of MMP1 (**F**) and MMP3 (**G**) expression based on mean DAB staining intensity. Quantification was performed using five randomly selected dermal fields per group. All quantitative data represent mean ± SD from *n* = 5 animals per group. Statistical analysis was performed using the Kruskal–Wallis test followed by the Mann–Whitney U test for post hoc comparison. Bars labeled with different letters indicate statistically significant differences between groups (*p* < 0.05). ELISA, enzyme-linked immunosorbent assay; IHC, immunohistochemical staining; MMP, matrix metalloproteinases; PLLA, poly-l-lactic acid; SD, standard deviation; W, weeks.

**Figure 5:**
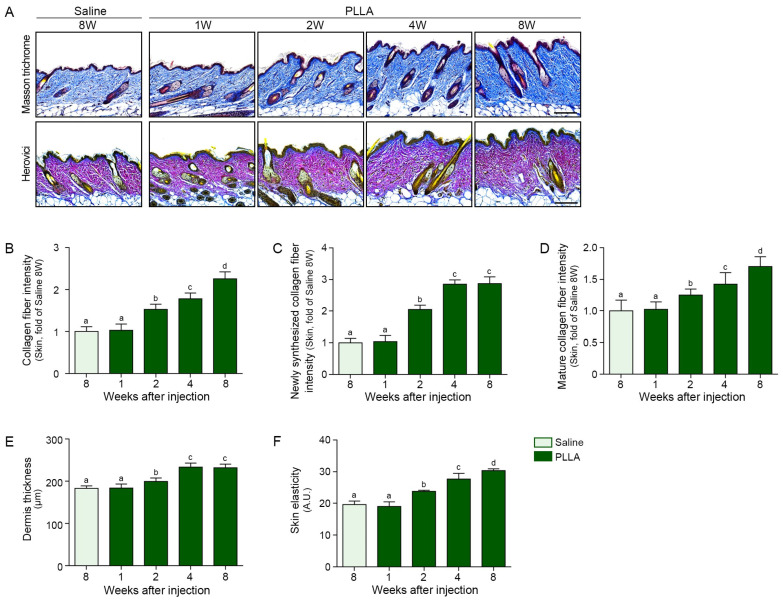
PLLA injection enhances collagen fiber density, dermal thickness, and skin elasticity in aged mouse skin. (**A**) Representative histological images of skin sections stained with Masson trichrome (upper panels) to visualize total collagen (blue) and Herovici stain (lower panels) to distinguish newly synthesized collagen (blue) from mature collagen (red) (scale bar = 100 μm). (**B**) Quantification of total collagen fiber density based on Masson trichrome staining. (**C**,**D**) Quantification of newly synthesized collagen fibers (**C**) and mature collagen fibers (**D**) from Herovici-stained tissue. (**E**) Measurement of dermal thickness. (**F**) Skin elasticity was assessed using a cutometer. All quantitative data represent mean ± SD from *n* = 5 animals per group. Statistical analysis was performed using the Kruskal–Wallis test followed by the Mann–Whitney U test for post hoc comparison. Bars labeled with different letters indicate statistically significant differences between groups (*p* < 0.05). PLLA, poly-l-lactic acid; SD, standard deviation; W, weeks.

PLLA injection increased adiponectin expression over time in the aged SAT (Figure 4A) as well as collagen1 and 3 in the dermis of aged skin (Figure 4B–D). In contrast, PLLA decreased MMP1/3 over time in the dermis of the aged skin (Figure 4E–G). The collagen fiber density in the dermis, evaluated by Masson trichrome staining, increased over time after PLLA injection (Figure 5A,B). Newly synthesized collagen, which stains blue by Herovici staining versus red for mature collagen [49,50], increased up to 4 weeks after PLLA injection (Figure 5A,C). PLLA treatment also resulted in an increase in mature collagen over time in the dermis of the aged skin (Figure 5A,D). Similarly, dermal thickness increased up to 4 weeks after PLLA injection and skin elasticity, measured using a portable skin analyzer, also increased over time after PLLA treatment (Figure 5E,F).

## 4. Discussion

In contrast to other adipose tissues, such as visceral fat, SAT decreases in volume and thins during aging [51]. It plays a key role in maintaining a youthful facial appearance and provides important physiological functions by secreting adipokines and communicating with dermal and immune cells [52]. Also, SAT serves as a niche for precursor and stem cells [52]. However, during aging, the loss of these cells leads to a decline in adipokine secretion due to reduced adipogenesis [51,53]. Consequently, SAT not only diminishes in volume but also becomes dysfunctional over time. Various methods have been tested to restore SAT during aging by increasing adipogenesis from precursors or adipose-derived stem cells (ADSCs) [51]. HA filler increases the proliferation of ADSCs and differentiation to adipocytes [2,54,55,56]. HA filler also decreases lipolysis and increases adiponectin during the differentiation of preadipocyte to adipocyte [55] and stimulates ADSCs to increase adipose tissue volume [54].

Notably, adipogenesis is beneficial only when the newly generated adipose tissue is not dysfunctional. Increased adipose tissue volume or thickness occurs by hyperplasia (increasing cell number) and hypertrophy (increasing cell size) [57]. However, hypertrophic adipocytes—commonly associated with obesity—produce inflammatory cytokines that jeopardize metabolic health. Moreover, hypertrophic adipocytes secrete less adiponectin, which inhibits collagen and elastin genes and promotes MMP13 and MMP9 in fibroblasts in adipocytes [58,59]. They release higher levels of free fatty acids, such as palmitic acid, which raise lipotoxicity in fibroblasts and contribute to ECM degradation [58]. Therefore, to support healthy adipose tissue expansion, it is crucial to prevent adipocyte hypertrophy and the associated decline in adiponectin secretion.

Previous studies evaluating the effects of fillers on adipose tissue expansion focused on their ability to promote the proliferation or adipogenesis of ADSCs or preadipocytes [54,55,56]. However, effective skin rejuvenation depends not only on adipose tissue expansion but also on the functionality of the tissue—particularly its ability to secrete adequate levels of adiponectin. Therefore, in this study, we determined whether PLLA enhances adiponectin secretion by supporting the maintenance of the mature adipocyte phenotype and appropriate cell size, as well as promoting adipogenesis.

NRF2 promotes adipogenesis by upregulating C/EBPα and PPARγ [32,33]. In an *NRF2* knockout, adipocyte differentiation decreases along with the downregulation of C/EBPα and PPARγ [32]. Loss of NRF2 by gene deletion or chemical inhibition decreases adipogenesis in 3T3-L1 preadipocytes or human subcutaneous preadipocytes [32,60].

In this study, we treated cells with H_2_O_2_ during the differentiation of preadipocytes into mature adipocytes to mimic the aging-related adipose tissue environment, where adipogenesis of adipocyte precursors is reduced. The 3T3-L1 cell line is a reliable model for evaluating adipogenesis from the preadipocyte to the mature adipocyte [61,62]. Differentiated 3T3-L1 preadipocytes are very similar in ultrastructure to the adipocytes of animal tissue [61], and the development and shape of the lipid droplets in the differentiated 3T3-L1 preadipocytes are similar to living adipose tissue [62].

The differentiation of 3T3-L1 cells is initiated by treatment with DM and insulin for two days and then maintained with insulin for the next six days to generate terminal mature adipocytes [63]. Our H_2_O_2_ treatment of cells at 4 days after the initiation of differentiation could inhibit the differentiation to mature adipocytes. Treatment with H_2_O_2_ increased senescence markers p16 and p21 in the adipocyte, and then treatment with PLLA increased expression of NRF2, C/EBPα, and PPARγ in the senescent adipocyte. PLLA also increased expression of the adipogenic markers FABP4, LPL, and CD36, as well as lipogenesis markers ACL, ACC, and FAS in the senescent adipocyte. Further, PLLA increased lipid droplet-laden cells. Together, these findings suggested that PLLA increased adipogenesis and lipogenesis in the senescent adipocyte. Although PLLA enhanced lipid storage in adipocytes along with increased NRF2 expression, we could not confirm a direct role of NRF2 in the upregulation of C/EBPα and PPARγ, as we did not inhibit NRF2 or delete the gene. The connection between NRF2 and C/EBPα and PPARγ in the PLLA-treated adipocyte should be evaluated further.

We found that PLLA increased adiponectin secretion from senescent adipocytes, and collagen1 and 3 increased when CM from PLLA-treated senescent adipocytes was added to senescent fibroblasts. In contrast, the expression of MMP1/3 decreased in the senescent fibroblast. These results suggest that PLLA promotes collagen synthesis and reduces ECM destruction in the fibroblast by modulating adipocytes. Further, adiponectin may play a key role in this adipocyte–fibroblast interaction.

In old mice, PLLA treatment increased expression of NRF2, C/EBPα, and PPARγ as well as adipogenic markers FABP4, LPL, and CD36 and lipogenesis markers ACL, ACC, and FAS in the aged SAT. We demonstrated that PLLA treatment increased the number of adipocytes without affecting their size and enhanced the expression of adiponectin, which is associated with increased adipogenesis in aged SAT.

Adiponectin plays a crucial role in adipocyte differentiation, increasing adipocyte number while decreasing adipocyte size [64]. Additionally, adiponectin secretion is negatively associated with adipocyte hypertrophy [65], as both adipocyte senescence and hypertrophy contribute to decreased adipogenesis [66]. Therefore, maintaining adiponectin secretion is necessary to increase adipocyte number without hyperplasia, which is linked to adipose tissue dysfunction. Our findings suggest that PLLA promotes adipogenesis and hyperplasia rather than hypertrophy.

Collagen1 and 3 are the most abundant types of collagen in the dermis [67,68,69]. Collagen1 is a structural fiber protein that maintains the mechanical strength of skin [67,70]. Collagen 3 forms fibrils with collagen1, providing skin elasticity [71]. Both types of collagen decrease in aging [72] with a reduction in collagen fibers, fragmentation of collagen, and a decrease in cell-to-collagen fiber interactions [73,74,75]. Destruction of collagen fibers and a decrease in the amount of collagen fibers reduce the structural support of the skin, which could lead to loss of volume and wrinkle formation [76]. Further, the expression of ECM-degrading enzymes, such as MMP, increases with age [77].

Previous studies showed that PLLA increases dermal thickness by stimulating collagen synthesis in the fibroblast [78]. Immune cells such as macrophages recognize PLLA as foreign bodies and stimulate a mild immune response that causes fibroblasts to synthesize collagen [79,80]. PLLA subcutaneous injection led to an increased inflammatory reaction, which was associated with elevated expression of tumor necrosis factor-alpha (TNF-α), interleukin-12 (IL-12), and TGF-β in rats at 12 weeks [81]. Collagen synthesis began to increase one week after injection and continued to rise up to 12 weeks. In this study, we did not evaluate the inflammatory reaction following PLLA injection, as we focused on the effect of PLLA on adiponectin, which may promote collagen accumulation rather than inflammation.

We found that collagen1 and 3 expressions increased, and MMP1/3 decreased after PLLA treatment of the skin. Additionally, Masson trichrome staining revealed an increase in collagen fiber density, including both newly synthesized and mature collagen fibers. PLLA also enhanced dermal thickness and skin elasticity. Our findings indicate that PLLA promotes collagen accumulation and improves skin elasticity, resulting in skin rejuvenation in aged skin.

The PLLA-induced increase in collagen synthesis and decrease in collagen destruction in aged skin was maintained for up to 8 weeks after injection. As well as increasing collagen accumulation, PLLA also induced adipogenesis and lipogenesis without adipocyte hypertrophy, which was accompanied by increased NRF2, C/EBPα, and PPARγ. Although previous studies demonstrated that fillers induce adipogenesis [54,55,56], our study showed that PLLA increased adiponectin, which induced adipogenesis without hypertrophy. Thus, PLLA could rejuvenate skin by increasing collagen accumulation which relates to increasing adiponectin in the adipose tissue.

This study has several limitations. Our study does not provide a mechanism by which PLLA increases NRF2, nor could we demonstrate that NRF2 directly upregulated C/EBPα and PPARγ after PLLA was injected in the aged SAT. These mechanisms should be determined in future studies. In this study, we hypothesized that adiponectin stimulates fibroblasts to promote collagen production. We confirmed that adiponectin levels in adipocyte culture media increase upon PLLA treatment, and, subsequently, treatment of fibroblasts with this conditioned media led to increased collagen synthesis. However, to determine whether the increase in fibroblast collagen synthesis is primarily due to adiponectin upregulation by PLLA, it is necessary to compare with a group in which adiponectin is directly applied to fibroblasts. A previous study reported that direct treatment of adipocytes with PLLA following UV irradiation resulted in increased expression of collagen IV mRNA in adipocytes [82]. This finding suggests that, in addition to enhancing adiponectin, PLLA may promote collagen synthesis through multiple pathways.

In this study, we did not assess whether collagen production persists beyond eight weeks following PLLA injection; therefore, future research should determine the duration of sustained collagen synthesis after PLLA administration. In particular, as the capacity for collagen synthesis may differ between young and aged skin [83], it is necessary to investigate the longevity of collagen production and the increase in SAT thickness induced by PLLA injection in aged animal models.

Additionally, while previous studies primarily focused on dermal injection of PLLA [41,84], in this study, PLLA was administered into the SAT layer to evaluate its specific effects on this compartment [85]. Future investigations should compare the extent of collagen synthesis between subcutaneous and dermal injection routes. If differences in collagen synthesis are observed depending on the injection method, the choice of PLLA administration technique should be tailored to the desired clinical outcome—whether the primary goal is to enhance collagen synthesis alone or to increase SAT thickness as well. Although the safety of PLLA has been well established in numerous preclinical and clinical studies [41], we did not evaluate the long-term incidence of complications in this study; thus, future studies should assess the long-term effects and safety profile of PLLA injection. Moreover, further studies are required to determine whether PLLA increases skin rejuvenation via increasing adipogenesis and adiponectin in humans. Despite these limitations, our study provides novel evidence that, in addition to the previously reported mechanism of fibroblast stimulation and increased collagen synthesis by PLLA, adipocytes may also be stimulated by PLLA, leading to increased adiponectin secretion and subsequent collagen production.

## 5. Conclusions

PLLA increased the NRF2/PPARγ/C/EBPα pathway, adipogenesis, and lipogenesis factors in the senescent adipocyte and the aged SAT. PLLA increased adipocyte numbers and lipid droplet numbers of the senescent adipocyte without increasing size. Those changes were accompanied by increased adiponectin. In the senescent fibroblast and aged dermis, PLLA increased collagen1/3 with decreased MMP1/3. The resulting increase in adiponectin not only supports adipose tissue function but also acts in a paracrine fashion to enhance collagen production by dermal fibroblasts and suppress MMP, thereby contributing to skin rejuvenation. These findings have several important clinical and cosmetology applications. First, PLLA fillers could serve as a dual-action injectable therapy for age-related facial volume loss, not only restoring subcutaneous fullness but also improving skin quality through enhanced collagen synthesis. Second, the ability of PLLA to increase adiponectin and collagen may be leveraged in the treatment of atrophic scars, lipoatrophy, and other conditions characterized by reduced dermal or subcutaneous volume. Third, the paracrine effects of adipocyte-derived factors on fibroblast function suggest that PLLA injection could be utilized in advanced anti-aging protocols, potentially improving skin elasticity, reducing wrinkles, and promoting overall skin health. In conclusion, our results provide a mechanistic basis for the clinical use of PLLA in aesthetic procedures. By targeting both adipose tissue and dermal compartments, PLLA offers a comprehensive approach to facial rejuvenation and soft tissue restoration, with potential applications extending beyond traditional filler use to broader fields in cosmetology.

## Data Availability

All data are contained within this article.

## Supplementary Materials

The following supporting information can be downloaded at: https://www.mdpi.com/article/10.3390/polym17131826/s1, Figure S1: Schematic overview of the experimental design for PLLA treatment in H_2_O_2_-induced senescent adipocytes.; Figure S2: Schematic overview of the experimental design for adipocyte-CM treatment in H_2_O_2_-induced senescent fibroblasts; Figure S3: In vivo experimental design for PLLA injection and sample collection in aged mice; Figure S4: Effects of PLLA on cell viability and *NRF2* expression; Figure S5: Quantification of adipogenic and lipogenic protein expression in SAT following PLLA injection in aged mice; Figure S6: Subcutaneous injection of saline does not alter adipocyte size, number, or SAT thickness over time in aged mice; Table S1. List of primers for qRT-PCR; Table S2. List of antibodies used for Western blot, ICC and IHC.
