# Supplementary material for: Poly-L-Lactic Acid Filler Increases Adipogenesis and Adiponectin in Aged Subcutaneous Tissue
Source: Polymers (Basel). 2025 Jun 30;17(13):1826. doi: 10.3390/polym17131826 (PMC12252205; doi:10.3390/polym17131826)
Supplement: Supplementary file 1 [file polymers-17-01826-s001.zip › polymers-3656701-supplementary.pdf]

polymers

Article

Poly-L-lactic acid filler increases adipogenesis and adiponectin in aged subcutaneous tissue

Seyeon Oh [1,2], Nala Shin [3], Sang Ju Lee [4], Kuk Hui Son [5,*] and Kyunghee Byun [1,6,7,*]

[1] Functional Cellular Networks Laboratory, Lee Gil Ya Cancer and Diabetes Institute, Gachon University, Incheon 21999, Republic of Korea; seyeon8965@gmail.com (S.O.)
[2] LIBON Inc., Incheon 22006, Republic of Korea
[3] Soonsoo Dermatology & Antiaging Center, Jeonju 54969, Republic of Korea; sofarsogod0633@gmail.com (N.S.)
[4] Yonsei Star Skin & Laser Clinic, Seoul 03789, Republic of Korea; drderma@naver.com (S.J.L.)
[5] Department of Thoracic and Cardiovascular Surgery, Gachon University Gil Medical Center, Gachon University, Incheon 21565, Republic of Korea
[6] Department of Anatomy & Cell Biology, College of Medicine, Gachon University, Incheon 21936, Republic of Korea
[7] Department of Health Sciences and Technology, Gachon Advanced Institute for Health & Sciences and Technology (GAIHST), Gachon University, Incheon 21999, Republic of Korea
* Correspondence: dr632@gachon.ac.kr (K.H.S.); khbyun1@gachon.ac.kr (K.B.); Tel.: +82-32-460-3666 (K.H.S.); +82-32-899-6511 (K.B.)

Academic Editor: Firstname Lastname

Received: date
Revised: date
Accepted: date
Published: date

Citation: To be added by editorial staff during production.

Copyright: © 2025 by the authors. Submitted for possible open access publication under the terms and conditions of the Creative Commons Attribution (CC BY) license (https://creativecommons.org/licenses/by/4.0/).

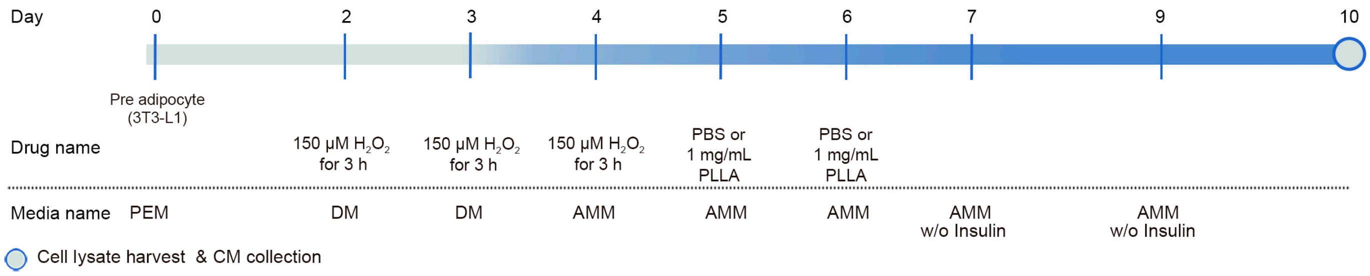

Figure S1. Schematic overview of the experimental design for PLLA treatment in H_2O_2-induced senescent adipocytes. 3T3-L1 preadipocytes were cultured to confluence and differentiated using a stepwise protocol from PID0 to PID10. Cellular senescence was induced by treatment with 150 μM H_2O_2 for 3 hours per day from PID2 to PID4. PLLA was applied on PID5 following dose optimization (0–2 mg/mL), and 1 mg/mL was used for all subsequent experiments. Cells were maintained under adipogenic conditions, and samples were collected at PID10. AMM, adipocyte maintenance medium; CM, conditioned medium; DM, differentiation medium; h, hour; PEM, preadipocyte expansion medium; PID, post-induction day; PLLA, poly-l-lactic acid; w/o, without.

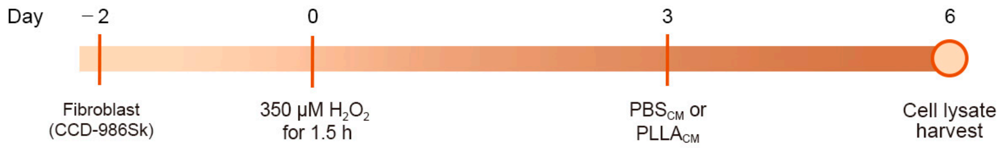

Figure S2. Schematic overview of the experimental design for adipocyte-CM treatment in H_2O_2-induced senescent fibroblasts. CM from adipocyte (PBS_{CM} or $PLLA_{CM}$) was applied to senescent fibroblasts. Fibroblasts (CCD-986Sk) were pretreated with 350 μM H_2O_2 for 1.5 hours, recovered for 72 hours, and then incubated with PBS_{CM} or $PLLA_{CM}$ for 3 days before analysis. CM, conditioned medium; h, hour.

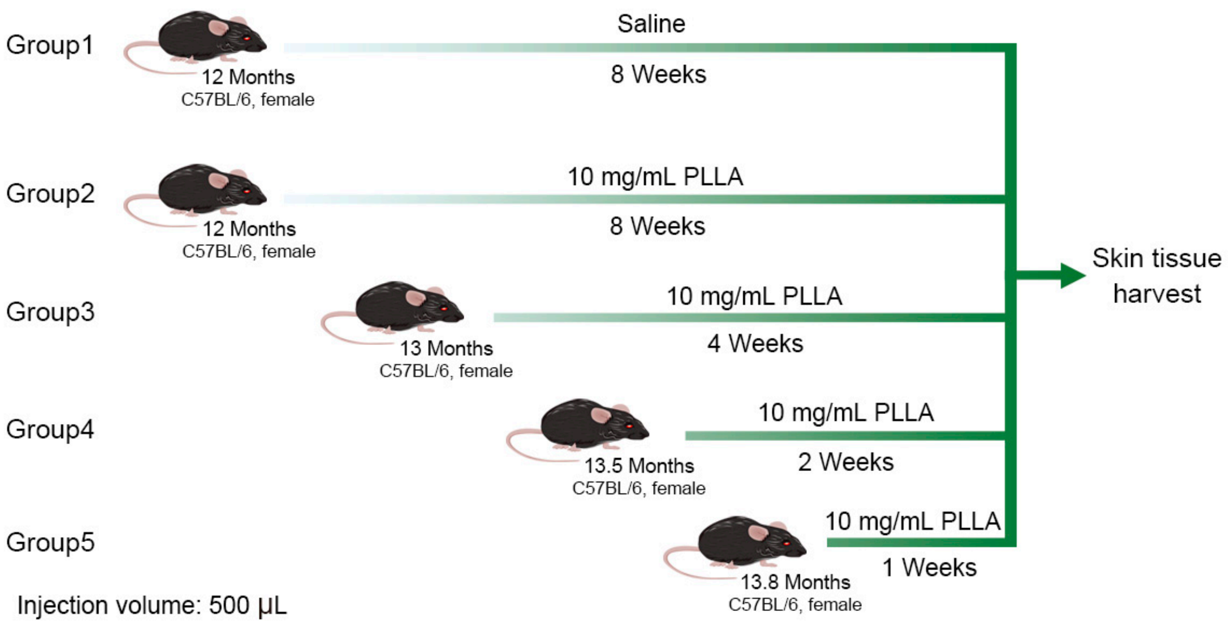

Figure S3. *In vivo* experimental design for PLLA injection and sample collection in aged mice. Female mice were randomly assigned to five groups (n = 5 per group) and received subcutaneous injections of either saline (Group 1) or PLLA (10 mg/mL, Group 2–5) into the back skin region. Injections were administered at five sites within a 2 cm × 2 cm area, with a total volume of 500 μL per mouse. To synchronize tissue collection at 14 months of age, we staggered the injection times: Groups 1 and 2 were injected at 12 months (harvested after 8 weeks), Group 3 at 13 months (4 weeks), Group 4 at 13.5 months (2 weeks), and Group 5 at 13.8 months (1 week). Skin and SAT samples were collected at the designated time points for analysis. PLLA, poly-l-lactic acid; SAT, subcutaneous adipose tissue.

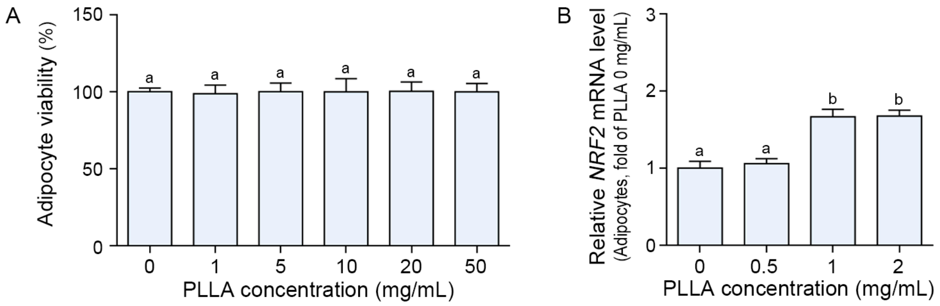

Figure S4. Effects of PLLA on cell viability and *NRF2* expression. (**A**) Cell viability was assessed using the CCK-8 assay after treatment of senescent adipocytes with increasing concentrations of PLLA (0–50 mg/mL). (**B**) Expression of *NRF2* was measured by qRT-PCR following PLLA treatment. All values are presented as the mean ± SD from at least three independent experiments. Statistical analysis was performed using the Kruskal–Wallis test followed by the Mann–Whitney U test for post hoc comparison. Bars labeled with different letters indicate statistically significant differences at $p < 0.05$. PLLA, poly-l-lactic acid; NRF2, nuclear factor erythroid-2-related factor 2; SD, standard deviation.

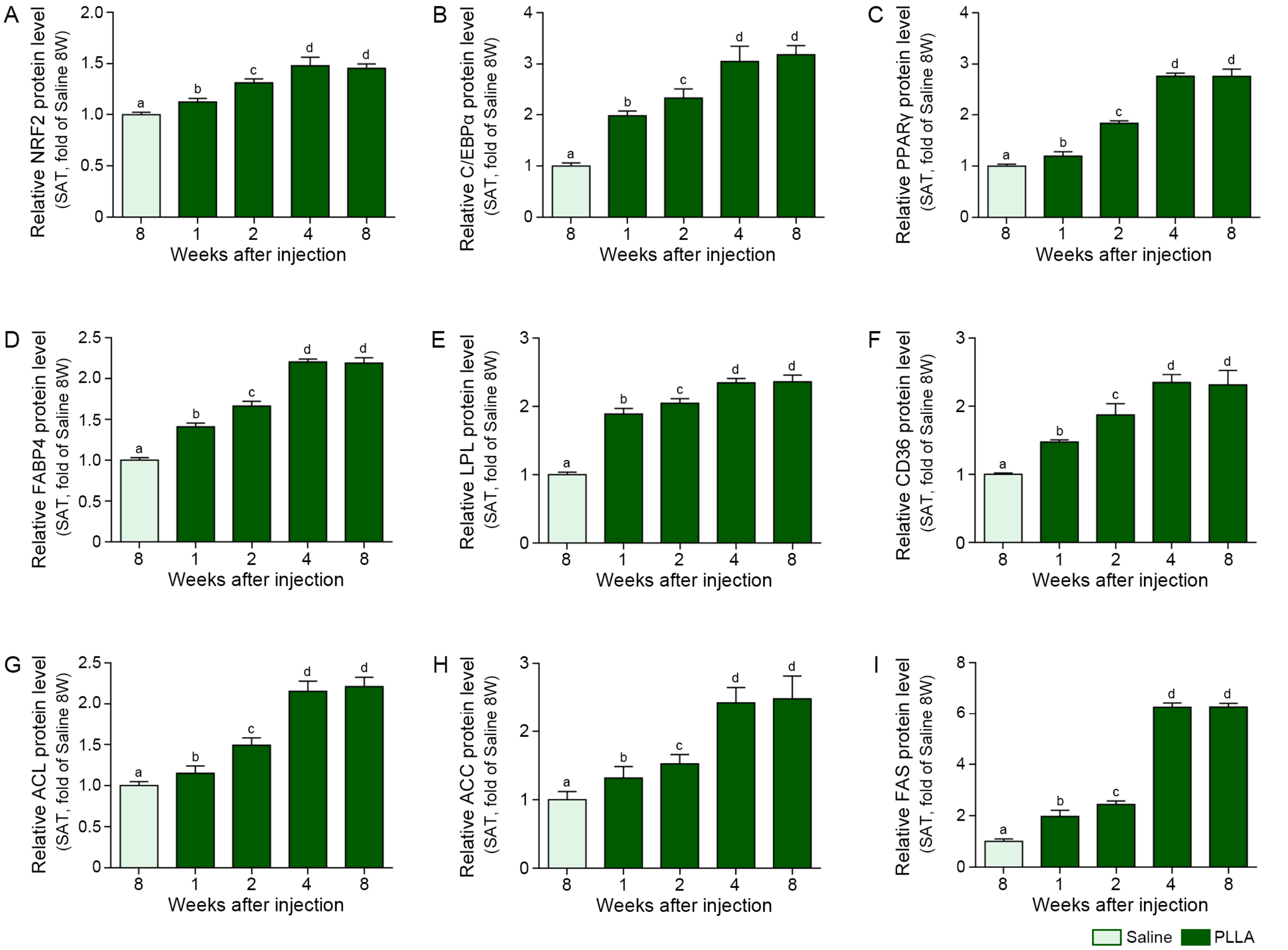

Figure S5. Quantification of adipogenic and lipogenic protein expression in SAT following PLLA injection in aged mice. (**A–C**) Relative protein expression levels of adipogenic transcription factors NRF2 (A), C/EBPα (B), and PPARγ (C). (**D–F**) Expression levels of adipogenic factors FABP4 (D), LPL (E), and CD36 (F). (**C-I**) Quantification of lipogenesis-related factors ACL (G), ACC (H), and FAS (I). Quantification of protein expression in (A), normalized to β-actin and calculated as fold change relative to Young. All quantitative data represent mean ± SD from n = 5 animals per group. Statistical analysis was performed using the Kruskal–Wallis test followed by the Mann–Whitney U test for post hoc comparison. Bars labeled with different letters indicate statistically significant differences between groups (p < 0.05). ACC, acetyl-CoA carboxylase; ACL, ATP citrate lyase; CD36, cluster of differentiation 36; C/EBPα, CCAAT/enhancer binding protein alpha; FABP4, fatty acid binding protein 4; FAS, fatty acid synthase; LPL, lipoprotein lipase; MW, molecular weight; NRF2, nuclear factor erythroid-2-related factor 2; PLLA, poly-l-lactic acid; PPARγ, peroxisome proliferator-activated receptor gamma; SAT, Subcutaneous adipose tissue; SD, standard deviation; W, weeks.

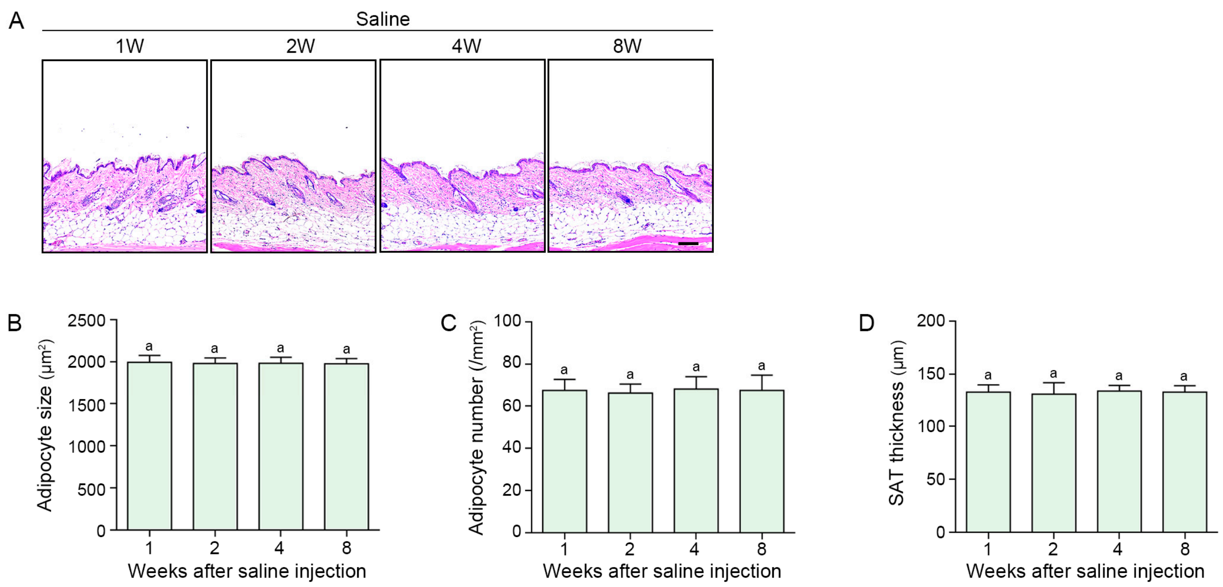

Figure S6. Subcutaneous injection of saline does not alter adipocyte size, number or SAT thickness over time in aged mice. (**A**) Representative H&E-stained images of skin tissue sections from mice injected with saline and harvested at 1-, 2-, 4-, and 8-weeks post-injection (scale bar = 100 μm). (**B–D**) Quantification of average adipocyte size (B), adipocyte number (C), and SAT thickness (D) at each time point. All quantitative data represent mean ± SD from $n = 5$ animals per group. Statistical analysis was performed using the Kruskal–Wallis test followed by the Mann–Whitney U test for post hoc comparison. Bars labeled with different letters indicate statistically significant differences between groups ($p < 0.05$). H&E, hematoxylin and eosin; SAT, Subcutaneous adipose tissue; SD, standard deviation; W, weeks.

Table S1. List of primers for qRT-PCR.

Gene (Organism)	Primer sequences	
actb (mouse)	Forward	5'-AAA GCC TAT TTC TGC CAG GAC-3′
	Reverse	5'-TCA TAG GTC ACG TAG CCC ACT-3′
p21 (mouse)	Forward	5'-ACC AGA GGC AGT AAC CAT GC-3′
	Reverse	5'-TGC TTC TAC AAA CCC ACA AAT G-3′
p16 (mouse)	Forward	5'-GGT TCT TGG TCA CTG TGA GGA T-3′
	Reverse	5'-GCA CGA ACT TCA CCA AGA AAA-3′
Nrf2(mouse)	Forward	5'-GCC CAG AAC TGT AGG AAA AGG A-3′
	Reverse	5'-TCT CTC GTC TTT TAA GTG GCC C-3′
ACTB (human)	Forward	5'-CTC GCC TTT GCC GAT CC-3′
	Reverse	5'-TCT CCA TGT CGT CCC AGT TG-3′
P21 (human)	Forward	5'-GGA GAC TCT CAG GGT CGA AAA-3′
	Reverse	5'-GCT TCC TCT TGG AGA AGA TCA G-3′
P16 (human)	Forward	5'-CAC TTT CCT GGG CAA CAA ATA-3′
	Reverse	5'-CTT GCG GTC ATC ATC GTA GTT-3′

The table presents the list of primer used in this study for qRT-PCR. Actb, actin beta; Nrf2, nuclear factor erythroid 2–related factor 2; qRT-PCR, quantitative reverse–transcription polymerase chain reaction.

Table S2. List of antibodies used for western blot, ICC and IHC.

Antibody	Dilution rate		
	Western blot	ICC	IHC
**ACC**	1:1,000		
**ACL**	1:1,000		
**β-actin**	1:1,000		
**C/EBPα**	1:1,000		
**CD36**	1:1,000		
**Collagen 1**		1:50	1:50
**Collagen 3**		1:100	1:200
**FABP4**	1:2,000		
**FAS**	1:400		
**LPL**	1:1,000		
**MMP1**	1:1,000		1:50
**MMP3**	1:1,000		1:200
**NRF2**	1:500		
**PPARγ**	1:400		

The table presents the list of antibodies used in this study for Western blot, ICC and IHC. ACC, acetyl-CoA carboxylase; ACL, ATP citrate lyase; CD36, cluster of differentiation 36; C/EBPα, CCAAT/enhancer binding protein alpha; FABP4, fatty acid binding protein 4; FAS, fatty acid synthase; ICC, Immunocytochemistry; IHC, Immunohistochemistry; LPL, lipoprotein lipase; MMP, matrix metalloproteinases; NRF2, nuclear factor erythroid-2-related factor 2; PPARγ, peroxisome proliferator-activated receptor gamma.
